# Laulimalide Induces Dose-Dependent Modulation of Microtubule Behaviour in the *C. elegans* Embryo

**DOI:** 10.1371/journal.pone.0071889

**Published:** 2013-08-02

**Authors:** Megha Bajaj, Martin Srayko

**Affiliations:** Department of Biological Sciences, University of Alberta, Edmonton, Alberta, Canada; Florida State University, United States of America

## Abstract

Laulimalide is a microtubule-binding drug that was originally isolated from marine sponges. High concentrations of laulimalide stabilize microtubules and inhibit cell division similarly to paclitaxel; however, there are important differences with respect to the nature of the specific cellular defects between these two drugs and their binding sites on the microtubule. In this study, we used *Caenorhabditis elegans* embryos to investigate the acute effects of laulimalide on microtubules *in vivo*, with a direct comparison to paclitaxel. We observed surprising dose-dependent effects for laulimalide, whereby microtubules were stabilized at concentrations above 100 nM, but destabilized at concentrations between 50 and 100 nM. Despite this behaviour at low concentrations, laulimalide acted synergistically with paclitaxel to stabilize microtubules when both drugs were used at sub-effective concentrations, consistent with observations of synergistic interactions between these two drugs in other systems. Our results indicate that laulimalide induces a concentration-dependent, biphasic change in microtubule polymer dynamics in the *C. elegans* embryo.

## Introduction

Microtubules are thin fibrous polymers that are crucial for cellular morphology, intracellular transport, cell motility and cell division. The polymers are composed of α/β tubulin heterodimer subunits that are added during polymerization (growth) or removed during depolymerization (shrinkage). A number of factors are required for the regulation of polymer dynamics to ensure that the microtubule fibers assemble precisely at the proper time and place [1–3]. Microtubules normally cycle between phases of growth and shrinkage and factors that control the balance between these phases help determine the long-term stability of individual polymers [2,4,5]. Many naturally occurring chemicals also bind to microtubules and influence microtubule dynamics, functioning as potent antimitotic toxins [6–8]. For this reason, microtubules continue to be one of the most effective targets for anticancer drugs.

Paclitaxel and related taxoids are widely used for treating a broad spectrum of tumors including ovarian, breast and lung carcinomas [9,10]. Mammalian cells treated with paclitaxel have extensive arrays of stabilized microtubule bundles and they usually arrest in the M phase of the cell cycle [8,11,12]. However, many tumors become resistant to paclitaxel and other antimitotic drugs [10,13]. Therefore, it is desirable to identify new microtubule targeting drugs that are effective on paclitaxel-resistant cell lines. In addition to their use in anticancer therapy, drugs with novel mechanisms of action and/or distinct microtubule binding sites could potentially identify relationships between specific subsets of cellular pathways and polymer ultrastructure.

Laulimalide is a naturally-occurring molecule originally isolated from the marine sponge *Cacospongiamycofijiensis* [14]. *In vivo* and *in vitro* studies indicate that laulimalide exhibits microtubule-stabilizing activity that is similar to paclitaxel [12] and it is considered another potent microtubule-targeting inhibitor of cellular proliferation. As with paclitaxel, laulimalide treatment results in an increased rate of apoptosis in a cell population [12,15,16]. Despite these similarities, there are also some important differences reported for paclitaxel and laulimalide. Mammalian cells treated with laulimalide or paclitaxel have abnormal mitotic spindles, however, the laulimalide-treated mitotic cells often exhibit a single radially-symmetric microtubule array surrounding a clear central core, whereas paclitaxel-treated cells have been shown to exhibit tetra- or tripolar spindles [14]. In addition, differences in the morphology of stabilized microtubule bundles in drug-treated cells have been reported. Paclitaxel resulted in long, thick microtubule bundles, whereas the laulimalide-induced microtubule bundles were short [14]. Evidence of increased kinetochore tension has been observed in mammalian cell lines treated with low nM doses of laulimalide, however, the reason for this effect is unclear [17].

Mass-shift perturbation studies have shown that laulimalide binds to β-tubulin, on the exterior of the microtubule lattice near the charged C-terminal tail [18]. This site is distinct from the paclitaxel-binding site [18–20]. Thus, laulimalide represents a class of microtubule stabilizer that has some similarities to paclitaxel but is also significantly different. Importantly, previous studies have focused on the long-term effects of laulimalide and thus acute effects have not been extensively investigated. Characterizing the cellular defects associated with laulimalide is an important step towards the development of this drug as an alternate microtubule-based cancer therapeutic.

Microtubule stabilization leading to cessation of cell division and eventual cell death depends on the concentration of the applied drug and the length of treatment [21]. Initial assays involving laulimalide at high concentrations and for lengthy incubation periods are likely to result in secondary phenotypes that might mask the underlying acute effects of the drug. In this study we use the rapidly dividing *Caenorhabditis elegans* one-cell embryo to investigate the acute effects of laulimalide on the first mitotic division. By comparison to the known microtubule-stabilizer paclitaxel, and the depolymerizing drug nocodazole, our results indicate that laulimalide-treated cells show striking dose-dependent phenotypes consistent with stabilizing microtubules at high concentrations and destabilizing microtubules at lower concentrations. The cellular phenotypes associated with microtubule destabilization at low laulimalide concentrations is an important distinction from the cellular phenotypes observed with paclitaxel. We also show that doses of laulimalide that induce depolymerization can still enhance the stabilization of microtubules when applied in combination with low doses of paclitaxel. This result is consistent with previous reports that paclitaxel and laulimalide can act synergistically [22].

## Materials and Methods

### Permeabilizing the egg shell

In order to generate permeable embryos, *perm-1*(*RNAi*) or *ptr-2*(*RNAi*) was used [23]. A bacterial clone expressing *perm-1* or *ptr-2* dsRNA was grown in Luria Broth with 50 µg/mL ampicillin at 37 ^°^C for 10-12 hours [24]. The next day an RNAi plate of nematode growth medium (NGM) agar containing 1 mM IPTG and 25 µg/mL carbenicillin was seeded with the overnight culture (~300 µL). The plates were allowed to dry at room temperature for 7-8 hours. 20-30 L4 worms were transferred to the seeded NGM agar plate and incubated at 20 ^°^C for 10-12 hours. The fluorescent strain MAS91 *unc-119*(*ed3*)*;* [*pie-1::gfp::beta-tubulin, unc-119(+)*]*;* [*pie-1::mcherry::histone, unc-119(+)*] was made by crossing strains AZ244 and OD83, and was used in all live-cell microscopy experiments.

### Drug delivery and live-cell imaging

For drug delivery, 2 worms were dissected in a drop of 5 µL Egg Buffer (188 mM NaCl, 48 mM KCl, 2 mM CaCl_2_, 2 mM MgCl_2_, and 25 mM HEPES, pH 7.3) mounted on a L-polylysine (Sigma-Aldrich #P8920) coated coverslip. Coverslips were covered with a removable transparent chamber during image acquisition to prevent evaporation. Embryos in early prophase were first imaged in 5 µL egg buffer, without drug. After pronuclear centering, 5 µL of drug (in egg buffer + DMSO) was added to the egg buffer on the polylysine-coated coverslip to achieve the final drug concentration. The final concentration of DMSO did not exceed 5% for all drug treatments. To ensure that the observed phenotypes were not due to DMSO, all control samples were treated similarly with 5% DMSO. After drug addition, filming of the embryo was continued up to the end of the first mitotic division for most of the embryos. Paclitaxel (Sigma # T7191), nocodazole (Sigma # M1404) and laulimalide (gift from Dr. David Schriemer, University of Calgary) dissolved in DMSO were used at varying concentrations for experiments.

### Immunostaining and microscopy

*ptr-2*(*RNAi*) was used to permeabilize wild-type N2 embryos as described above. Gravid worms were placed into 5 µL of the appropriate drug solution on poly-lysine coated slides. A coverslip was then placed on these worms and gentle pressure was applied on the coverslip to release the embryos into the solution. The released embryos were incubated in the drug for 5 minutes. Slides were then plunged into liquid nitrogen for 10 minutes. Each slide was retrieved and the coverslip flicked off while still frozen, and the slide was put into -20 °C methanol for 15 minutes. Slides were washed twice in a coplin jar containing phosphate-buffered saline (PBS), and a total volume of 100 µL of primary antibody (in PBS with 5% goat serum, 0.01% Triton X-100) was pipetted onto the fixed embryos. Slides were incubated at room temperature in a humid chamber for 45 minutes, followed by 2 x 10 minute PBS washes in a coplin jar. Secondary antibodies were applied using the same method. Embryos were stained with DAPI (1 µg/mL) and mounted with glycerol-PPD media (90% glycerol, 20mM Tris-HCl, pH 8.8, 0.5% p-phenylenediamine). Coverslips were sealed with nail polish. Primary mouse anti-tubulin (DM1A; 10 µg/mL; Sigma) and rabbit-anti-TBG-1 (35 µg/mL) [25] and secondary fluorescent anti-mouse Alexa 488 and anti-rabbit Alexa 546 (Invitrogen) antibodies were used in the experiment. DAPI was used to visualize the chromatin.

Imaging was performed with an Olympus inverted microscope (IX81; Olympus, 60x oil objective NA 1.42) with a spinning disc confocal head (CSU10; Yokogawa) modified with a condenser lens in the optical path (Quorum Technologies). All images were acquired with an ORCA-R2 camera (Hamamatsu) controlled by Metamorph software (Molecular Devices). For live-cell imaging of GFP-tubulin and mCherry-histone, two-colour Z-series stacks (5 planes at 2 µm spacing) were acquired (250 millisecond exposures; 2x2 binning) every 15 seconds. For immunostaining experiments, Z-series stacks (61 planes at 0.2 µm spacing) were acquired (no binning) for the control and experimental images using equivalent exposure times, within the dynamic range of the camera. Maximum projections of image stacks were generated for the whole embryo using false colors for display. Image analysis was done using Metamorph software.

## Results and Discussion

**Figure 1:**
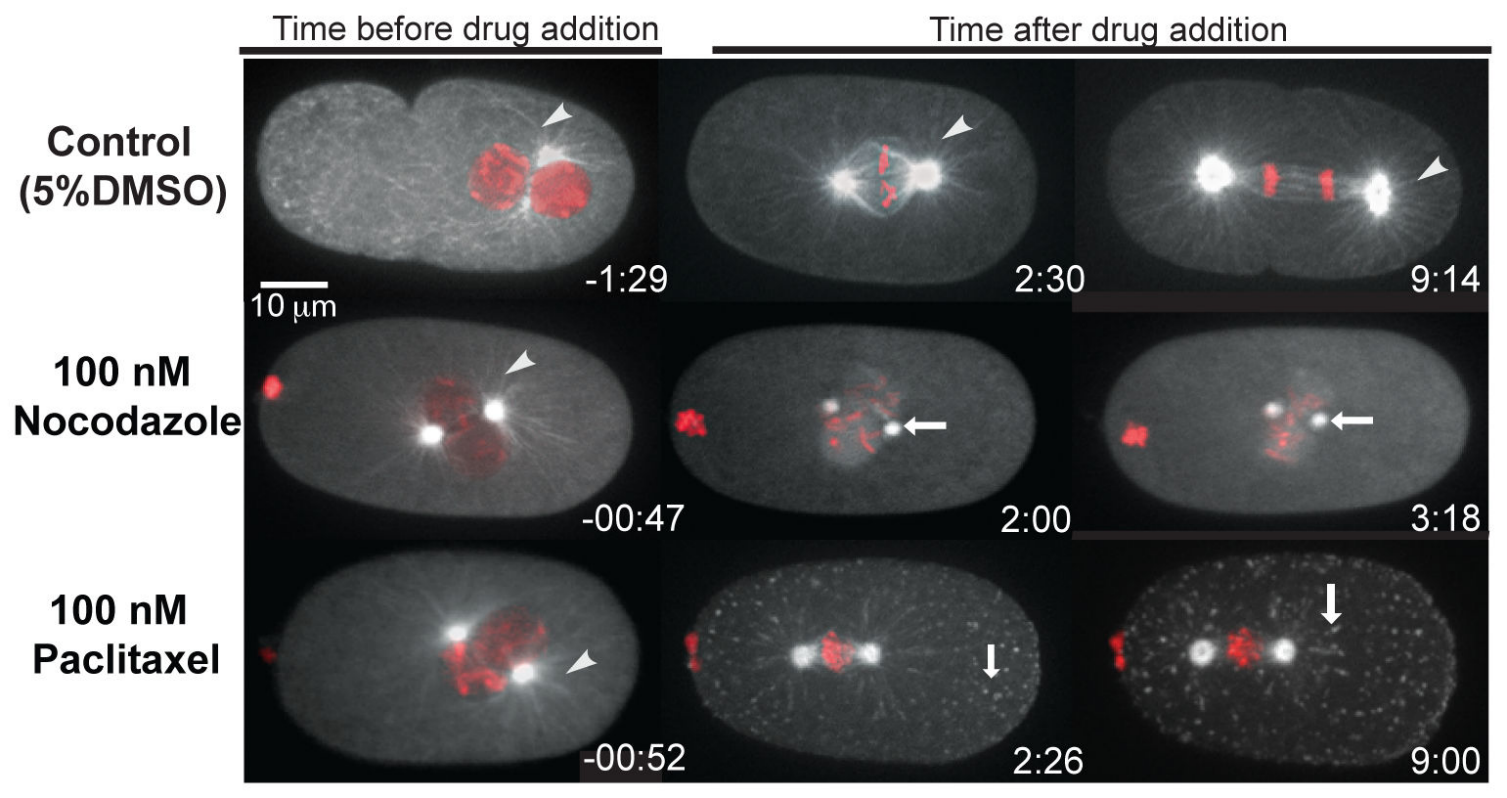
Drug tests on permeabilized embryos. Confocal images of embryos expressing GFP-tubulin and mCherry-histone are shown. The posterior of the embryos are to the right. The control embryo treated with 5% DMSO divided normally. The white arrowhead points to the microtubule fibres, which were observed in all embryos before drug addition. Embryos treated with nocodazole and paclitaxel did not divide and microtubule fibres were seen in the time frame before drug addition indicated by white arrowheads. In nocodazole-treated embryo, fluorescence was visible only at the centrosomes (white arrows) and the centrosomes gradually drifted towards each other. In paclitaxel treated embryos, white arrows indicate tubulin aggregates in the cytoplasm.

In order to compare the effects of laulimalide and paclitaxel on microtubule-dependent processes during cell division we applied each chemical to early one-cell C*. elegans* embryos. The first mitotic division in *C. elegans* takes about 20 minutes for completion. In a specialized mitotic prophase, the female and male pronuclei initially form and then meet near the center of the embryo. This is followed by nuclear envelope breakdown (NEBD), mitotic spindle assembly, anaphase, and cytokinesis ( [26], Movie S1). *C. elegans* embryos develop within an eggshell that is impermeable to many drugs. In order to permeabilize the embryos, we used either *perm-1*(*RNAi*) or *ptr-2*(*RNAi*), both of which result in increased permeability of the eggshell to many compounds [23]. Because the microtubule-based processes in the one-cell embryo are highly stereotypical, drug application to embryos at a specific stage of the cell cycle provides a rapid and reliable method to identify distinct phenotypes resulting from different microtubule inhibitors. This makes the *C. elegans* one-cell embryo an attractive model for testing the acute effect of drugs. To first establish the phenotypic consequences of microtubule stabilization vs. destabilization on cell division processes in *C. elegans*, we applied paclitaxel (Movie S2) or nocodazole (Movie S3), at the pronuclear migration/pronuclear centering stage of mitosis. As shown in Figure 1, nocodazole resulted in the loss of centrosome-based microtubule fibres (shown by white arrows) within about 2 minutes of drug application (n = 15). Although a weak fluorescence signal remained at the centrosomes, spindle assembly was not observed and the embryos did not progress through the cell cycle (Figure 1). In contrast, paclitaxel-treated embryos exhibited defects consistent with the stabilization of microtubules and many fluorescent foci were observed in the cytoplasm. The foci did not display the same dynamic microtubule growth/shrinkage behaviour characteristic of astral microtubules, therefore, we refer to these as tubulin aggregates (n = 20; Movie S2; Figure 1; white arrowheads). The paclitaxel-treated embryos also did not assemble a proper mitotic spindle, and the cell cycle did not progress. The effects of nocodazole and paclitaxel treatment on microtubules in the *C. elegans* embryo were consistent with previous reports [23,27–29].

### Laulimalide destabilizes microtubules at low concentrations

**Figure 2:**
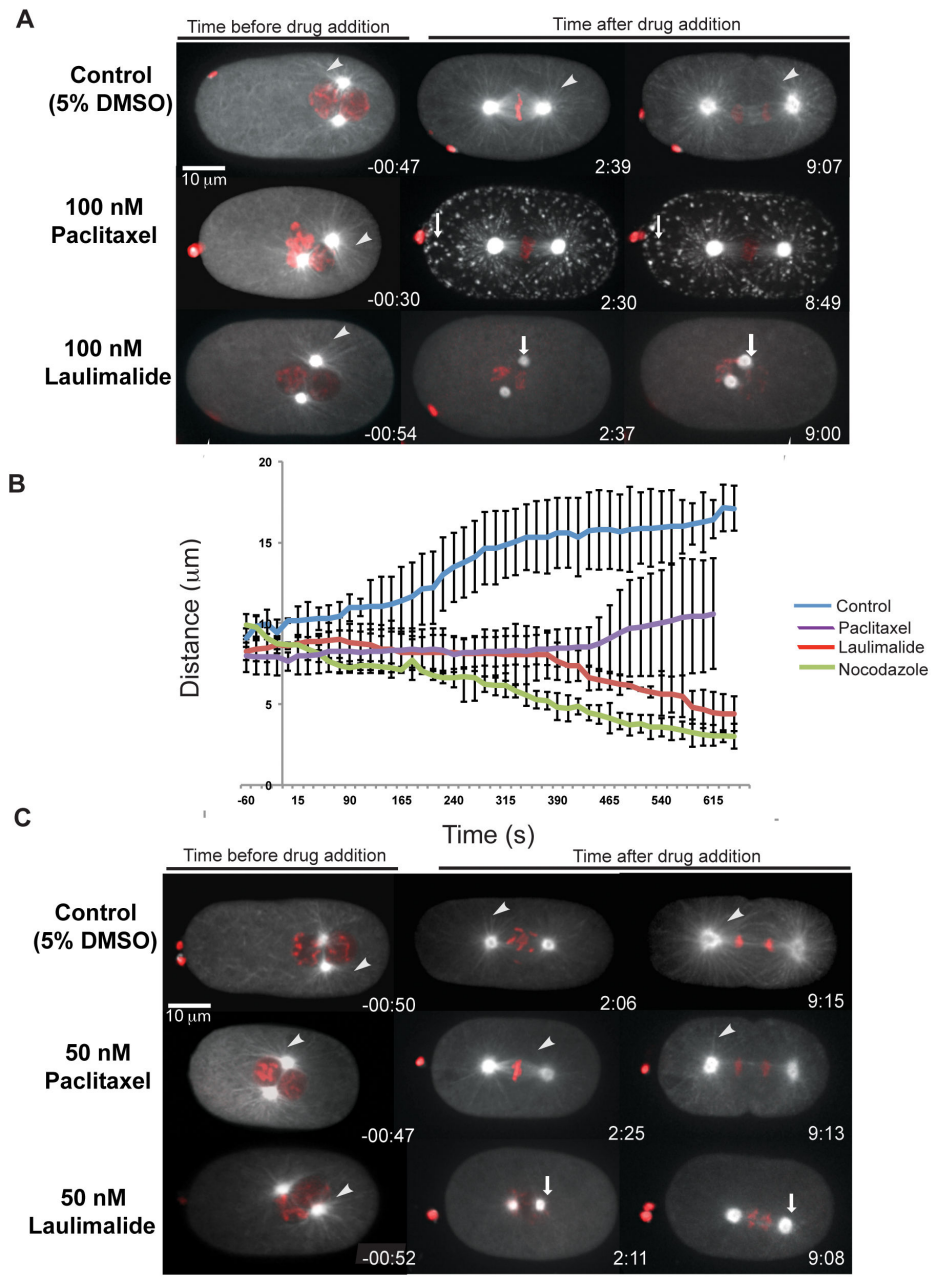
Laulimalide destabilizes microtubules at low concentration. Confocal images of embryos expressing GFP-tubulin and mCherry-histone were treated with 5% DMSO (control), paclitaxel, or laulimalide, as shown. The posterior of the embryos is to the right. The white arrowhead points to the microtubule fibres, which were observed in all embryos before drug addition. A) Paclitaxel-treated embryos exhibit tubulin aggregates (white arrows). In 100 nM laulimalide-treated embryos, fluorescence was visible only at the centrosomes (white arrows) after drug addition B) A graph shows the distance between centrosomes in control embryos and embryos treated with laulimalide (100 nM), paclitaxel (100 nM), and nocodazole (100 nM). The data is an average of 3 embryos for each trial. Error bars indicate standard deviation. C) Control embryos and embryos treated with 50 nM paclitaxel divide normally. Laulimalide-treated embryos (50 nM) do not complete cell division and embryos displayed an increase in fluorescence intensity at the centrosomes and fewer microtubule fibres after drug addition (white arrows).

To assess its effect on microtubules, we tested laulimalide at 100 nM (Movie S4), the concentration at which paclitaxel showed strong microtubule-stabilization defects. In contrast to 100 nM paclitaxel treatment, embryos with 100 nM laulimalide exhibited defects that were more comparable to the microtubule destabilization phenotypes observed with nocodazole treatment (Figure 1). Within 2 minutes of drug application there were no microtubules or fluorescent foci visible in 82% of the laulimalide-treated embryos (n = 17). In these embryos only fluorescence at the centrosomes was visible after drug application (Figure 2A). In addition, within 2 minutes of laulimalide application, the anterior and posterior centrosomes drifted towards each other. In control embryos, anterior and posterior centrosomes initially maintained their relative positions during mitotic spindle assembly after nuclear envelope breakdown (NEBD). Then, centrosomes moved apart just before chromatid separation, and continued to separate throughout anaphase, reaching a maximum separation of 18 µm at the end of anaphase (Figure 2B). Similar to laulimalide treatment, nocodazole treatment resulted in centrosomes migrating towards each other, suggesting that microtubule depolymerization is specifically correlated with this type of centrosome movement (Figure 2B). The migration of anterior and posterior centrosomes towards each other was not observed in paclitaxel-treated embryos (Figure 2B).

Because we were using a transgenic β-tubulin GFP strain for the above experiments, it was possible that the microtubule depolymerization phenotypes we observed with 100 nM laulimalide were dependent on the GFP microtubules present in this strain. For example, the GFP tag itself might interfere with drug-microtubule binding. Alternatively, overexpression of this β-tubulin isotype could alter microtubule dynamics in a subtle way, resulting in unique phenotypes with low doses of some drugs such as laulimalide. To rule out these possibilities, we examined the microtubule cytoskeleton by immunostaining fixed permeabilized wild-type embryos. As with the live-cell imaging experiments, fixed embryos also displayed phenotypes consistent with microtubule depolymerization when pre-treated with 100 nM laulimalide or 100 nM nocodazole (Figure S1). As expected, microtubule stabilization was observed when fixed embryos were pre-treated with 100 nM paclitaxel. From these results, we concluded that the observed drug-induced microtubule phenotypes were not specific to the GFP-β-tubulin-expressing strain.

We also observed a similar microtubule destabilization phenotype in embryos treated with 50 nM laulimalide (Movie S5). However, only 58% of the embryos were obviously affected at this dose (n = 12), and these embryos exhibited phenotypes that appeared less severe than those treated with 100 nM laulimalide. For instance, of the embryos that were affected by 50 nM laulimalide, all progressed into anaphase, based on centrosome separation. However, chromosome segregation was not normal; chromosomes remained in the centre of the embryo, and cytokinesis did not occur (Figure 2C). We did not detect any obvious abnormalities in one-cell embryos treated with 40 nM laulimalide (data not shown) or 50 nM paclitaxel, suggesting slightly different binding affinities for these respective microtubule drugs on *C. elegans* microtubules *in vivo*.

### Laulimalide stabilizes microtubules at higher concentrations

**Figure 3:**
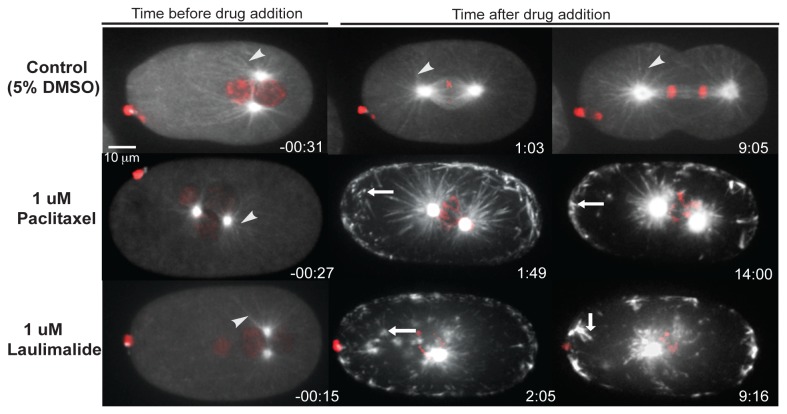
Laulimalide stabilizes microtubules at high concentration. Confocal images of embryos expressing GFP-tubulin and mCherry-histone are shown. The posterior of the embryos is to the right. Control embryo treated with 5% DMSO divides normally. The white arrowheads point to microtubule fibres. Microtubule fibres were present in all time frames in control embryo. Embryos treated with paclitaxel and laulimalide displayed prominent microtubule defects after drug addition. Spindle pole separation did not occur in these embryos. Tubulin aggregates in the cytoplasm (white arrows) were seen in both paclitaxel and laulimalide-treated embryos.

Laulimalide was previously shown to stabilize microtubules, however, the above experiments suggested that laulimalide could also cause destabilization of microtubules. The reason for microtubule destabilization could be due to its administration at low concentration. We therefore examined paclitaxel (Movie S6) and laulimalide (Movie S7) at the relatively high concentration of 1 µM. At this concentration application of either laulimalide or paclitaxel resulted in rapid stabilization of microtubules. Astral microtubule fibres ceased their characteristic dynamic movement and the centrosomal tubulin fluorescence appeared brighter. Furthermore, both drugs resulted in the formation of fluorescent foci throughout the cytoplasm that likely represented stabilized tubulin aggregates. These embryos did not complete the first cell division, but remained arrested at the stage at which the drug was administered. The chromosomes did not congress to form a metaphase plate (Figure 3).

**Figure 4:**
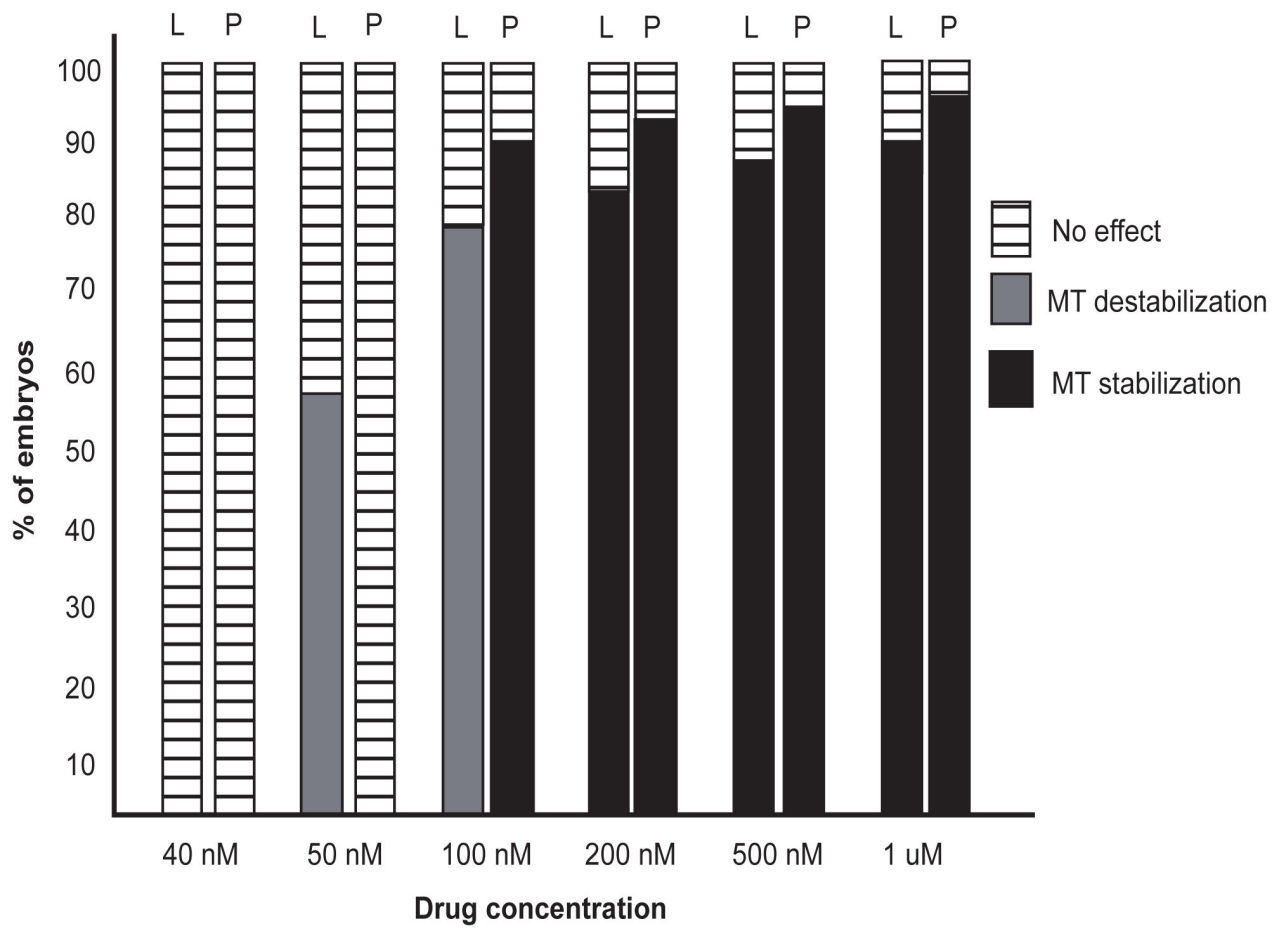
Dose response graph for laulimalide and paclitaxel. The graph shows the effect of various concentrations of drugs tested. n≥6 for each concentration used. L = laulimalide, P = paclitaxel.

Laulimalide and paclitaxel also resulted in embryonic phenotypes consistent with microtubule stabilization at intermediate concentrations of 500 nM and 200 nM (Figure 4). This indicated that, in *C. elegans* embryos, laulimalide acted as a microtubule stabilizing agent at concentrations of 200 nM to 1 µM, and a microtubule depolymerizer at concentrations of 50 to 100 nM (Figure 4).

### Synergistic effects of laulimalide and paclitaxel on microtubule dynamics

**Figure 5:**
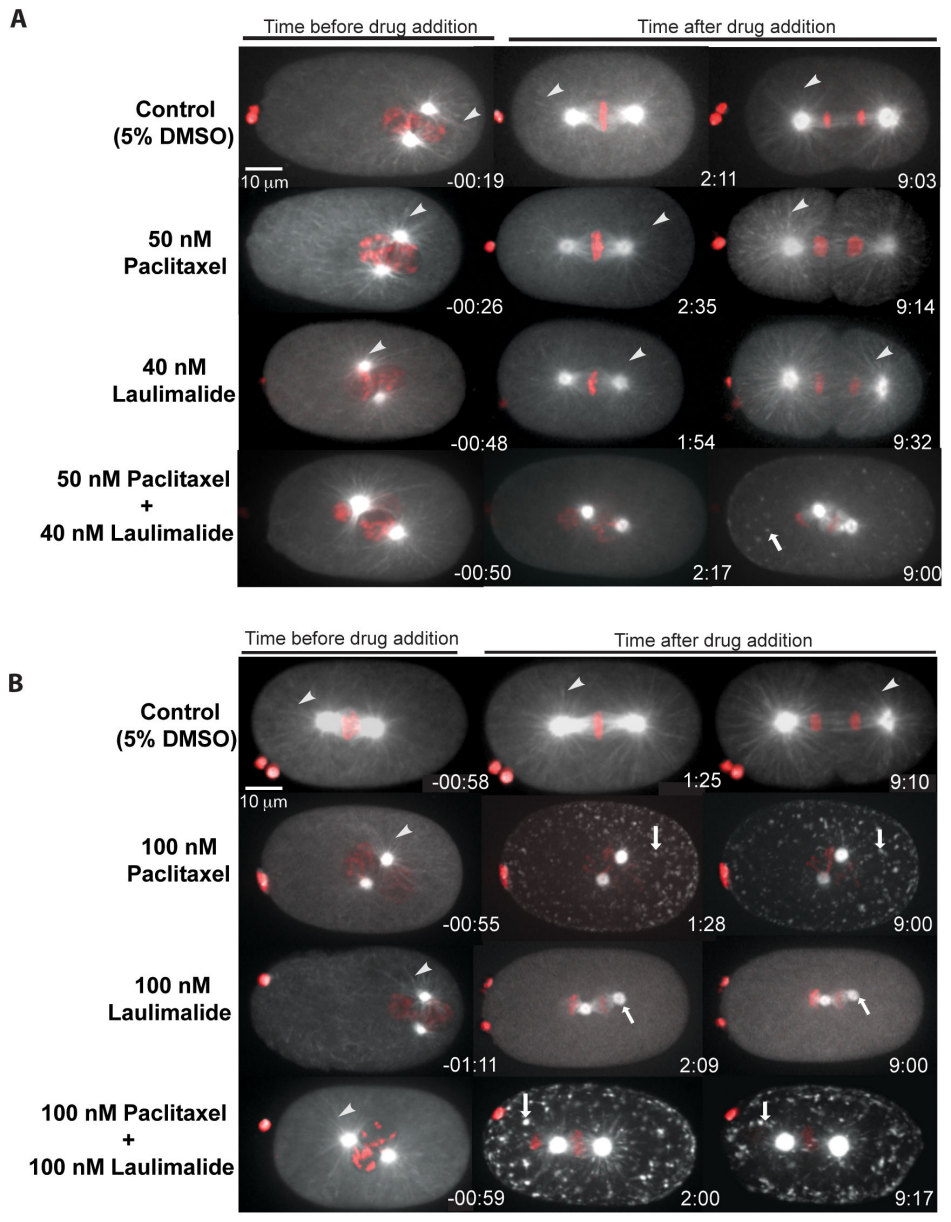
Laulimalide and paclitaxel act synergistically. A) Embryos treated with 5% DMSO (control), 50 nM paclitaxel or 40 nM laulimalide all exhibited normal cell division. White arrowheads indicate microtubule fibres, which were observed in all time frames. Embryos were also treated with a mixture 40 nM laulimalide and 50 nM paclitaxel, resulting in the appearance of tubulin aggregates (white arrows). A small spindle-like structure was observed, however, the morphology was abnormal and chromosome segregation did not occur. B) Embryos treated with 100 nM paclitaxel and 100 nM laulimalide show evidence of microtubule stabilization and destabilization respectively. Embryos treated with a mixture of laulimalide (100 nM) and paclitaxel (100 nM) displayed aggregates of tubulin (white arrows) and phenotypes similar to those observed with high doses of paclitaxel alone.

Laulimalide and paclitaxel have distinct binding sites on the α/β-tubulin heterodimer. Laulimalide binds on the exterior of microtubules on β-tubulin near the charged C-terminal tail [18]. Paclitaxel binds on the interior surface of microtubules within a pocket in the second globular domain of β-tubulin [30,31]. Distinct microtubule-binding sites for paclitaxel and laulimalide are consistent with synergistic effects that have been observed when both drugs were applied to either mammalian cells [22] or microtubules *in vitro* [32]. We tested embryos with a mixture of sub-effective concentrations of both drugs. When 40 nM laulimalide and 50 nM paclitaxel were used separately, microtubule dynamics were not affected and 100% of the tested embryos exhibited normal cell division (Figure 5A). However, when a combination of 40 nM laulimalide and 50 nM paclitaxel was used, spindle formation occurred but the spindle structure was not normal. Compared to the controls, these embryos had fewer microtubule fibres (Figure 5A). At about 2 minutes after drug application, fluorescent foci were present in the cytoplasm, as observed with high concentrations of laulimalide or paclitaxel. These embryos did not complete anaphase or undergo cell division (n = 8; Figure 5A). These results suggest that, at sub-effective concentrations, paclitaxel and laulimalide can act synergistically to perturb spindle structure and disrupt cell division in *C. elegans*.

We also tested for a synergistic effect of both drugs at 100 nM as each drug conferred different phenotypes at this concentration. This drug mixture resulted in phenotypes consistent with microtubule stabilization (as seen with 100 nM paclitaxel alone) in 83% of the tested embryos (n = 10). Stabilization of microtubules was observed within 2 minutes of drug application and the embryos did not recover even after 9 minutes after drug application. After 9 minutes, fewer microtubule fibres remained and stabilized foci were present in the cytoplasm (Figure 5B).

The microtubule-depolymerization phenotypes observed with low doses of laulimalide has not been previously reported. One possible reason is that the binding site targeted by laulimalide on *C. elegans* microtubules differs from that presented by microtubules from mammalian cell lines used in previous experiments. However, there are no differences between the *C. elegans* and bovine amino acid sequences within the critical region on β-tubulin that has been implicated in laulimalide binding [18]. This suggests that the microtubule depolymerization we observed at low laulimalide concentrations was likely not due to differences in the binding site for laulimalide on *C. elegans* β-tubulin. Microtubules purified from *C. elegans* exhibit an unusual 11-protofilament (pf) arrangement compared to canonical 13-pf mammalian microtubules [33], and this could alter polymer behaviour in the presence of small molecules. The 11-pf microtubule lattice might be more sensitive to low doses of laulimalide than the 13-pf counterpart. For instance, in situations where a small percentage of binding sites are occupied, 11-pf microtubules might become destabilized. At higher concentrations, the effect of multiple laulimalide molecules bound to multiple protofilaments of the same microtubule could instead stabilize the polymer. If the stabilization could be conferred by the occupation of either paclitaxel or laulimalide sites on the polymer, this would explain how low doses of paclitaxel can act synergistically with low doses of laulimalide to stabilize the microtubule.

The first embryonic cell mitosis takes only about 20 minutes to complete and microtubule polymerization rates are very fast at this stage (0.7 µm/sec) [34]. It is possible that the embryonic cellular environment contains a unique combination of microtubule-associated proteins (MAPs) and microtubule regulators that contribute to some of the laulimalide-induced phenotypes that we observed specifically in this system. Furthermore, if any cell-specific MAPs bind to the same site on the microtubule as laulimalide, this could cause unique responses in a cell-stage and/or tissue-specific manner.

In summary, our results indicate that the *C. elegans* microtubule network responds differentially to varying concentrations of laulimalide, compared with paclitaxel. Whether the difference in behaviour of the drugs is *C. elegans*-specific is not known at this time and further investigation is required. Our results also demonstrate that laulimalide and paclitaxel can act synergistically at sub-effective concentrations. It will be worthwhile to pursue such synergistic relationships between laulimalide and other antimitotic agents, for the development of potential combination-drug cancer therapies.

## Supporting Information

Figure S1 **Antibody staining of permeable fixed embryos.**
Representative control and drug-treated fixed embryos stained with anti-α tubulin and anti-γ tubulin antibodies to observe microtubules and centrosomes respectively, and DAPI to visualize chromatin.
(TIF)

Movie S1 **Permeabilized control embryo expressing GFP-Tubulin and mCherry-Histone.**
Treatment with 5% DMSO (final concentration) did not disrupt the microtubule network or the first cell division of permeabilized embryos.
(MOV)

Movie S2 **Permeabilized embryo expressing GFP-Tubulin and mCherry-Histone, treated with 100 nM paclitaxel.**
100 nM paclitaxel induced microtubule stabilization. Aggregates of tubulin were observed in the cytoplasm after drug addition (at time zero) and the cell did not divide.
(MOV)

Movie S3 **Permeabilized embryo expressing GFP-Tubulin and mCherry-Histone treated with 100 nM nocodazole.**
Addition of 100 nM nocodazole (at time zero) destabilized microtubules and the centrosomes drifted towards each other. The cell did not divide.
(MOV)

Movie S4 **Permeabilized embryo expressing GFP-Tubulin and mCherry-Histone treated with 100 nM laulimalide.**
Addition of 100 nM laulimalide (at time zero) destabilized microtubules and the centrosomes drifted towards each other. The cell did not divide.
(MOV)

Movie S5 **Permeabilized embryo expressing GFP-Tubulin and mCherry-Histone treated with 50 nM laulimalide.**
Addition of 50 nM laulimalide (at time zero) destabilized microtubules. The centrosomes moved apart consistent with anaphase initiation, however, chromosome segregation and cytokinesis did not occur.
(MOV)

Movie S6 **Permeabilized embryo expressing GFP-Tubulin and mCherry-Histone treated with 1 µM paclitaxel.**
Addition of 1 µM paclitaxel (at time zero) stabilized microtubules. Tubulin aggregates were visible in the embryo after drug addition. The cell did not divide and remained arrested at the stage at which the drug was administered.
(MOV)

Movie S7 **Permeabilized embryo expressing GFP-Tubulin and mCherry-Histone treated with 1 µM laulimaide.**
Addition of 1 µM laulimalide (at time zero) stabilized microtubules. Tubulin aggregates were visible in the embryo after drug addition. The cell did not divide and remained arrested at the stage at which the drug was administered.
(MOV)
