# Supplementary figures and images for: Laulimalide Induces Dose-Dependent Modulation of Microtubule Behaviour in the C. elegans Embryo
Source: PLoS One. 2013 Aug 2;8(8):e71889. doi: 10.1371/journal.pone.0071889 (PMC3732258; doi:10.1371/journal.pone.0071889)

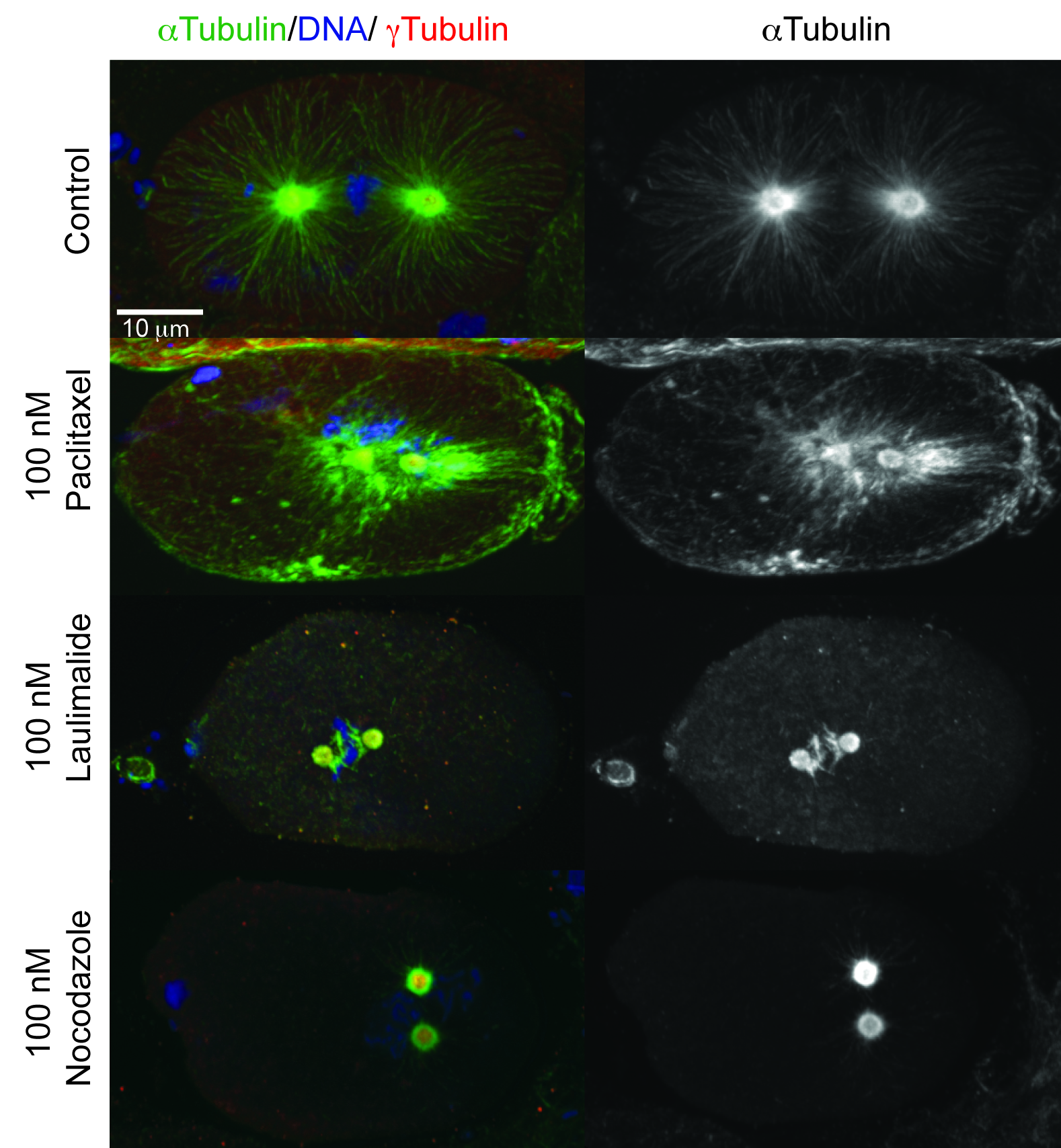

Supplement: Figure S1 — Antibody staining of permeable fixed embryos. Representative control and drug-treated fixed embryos stained with anti-α tubulin and anti-γ tubulin antibodies to observe microtubules and centrosomes respectively, and DAPI to visualize chromatin. (TIF) [file pone.0071889.s001.tif]
